# Supplementary material for: Histopathology images predict multi-omics aberrations and prognoses in colorectal cancer patients
Source: Nat Commun. 2023 Apr 13;14:2102. doi: 10.1038/s41467-023-37179-4 (PMC10102208; doi:10.1038/s41467-023-37179-4)
Supplement: Supplementary file 1 — Supplementary Information [file 41467_2023_37179_MOESM1_ESM.pdf]

Supplementary Information

Supplementary Figure 1. MOMA predicted survival outcomes of stage I and II colorectal cancer patients receiving surgery only, and stage III cancer patients without neoadjuvant therapy using digital histopathology images. (A) MOMA successfully distinguished the overall shorter-term survivors from longer-term survivors using histopathology images (two-sided log-rank test P-value = 0.015) of stage I and stage II patients receiving surgery only. (B) Among stage I and stage II patients without radiotherapy or chemotherapy, MOMA successfully distinguished progression-free survival groups using histopathology images (two-sided log-rank test P-value = 0.047). (C) MOMA successfully distinguished the overall shorter-term survivors from longer-term survivors using histopathology images (log-rank test P-value = 0.024) of stage III patients without neoadjuvant therapy. (D) Among stage III patients without neoadjuvant therapy, MOMA successfully distinguished the progression-free survival groups using histopathology images (log-rank test P-value = 0.018).

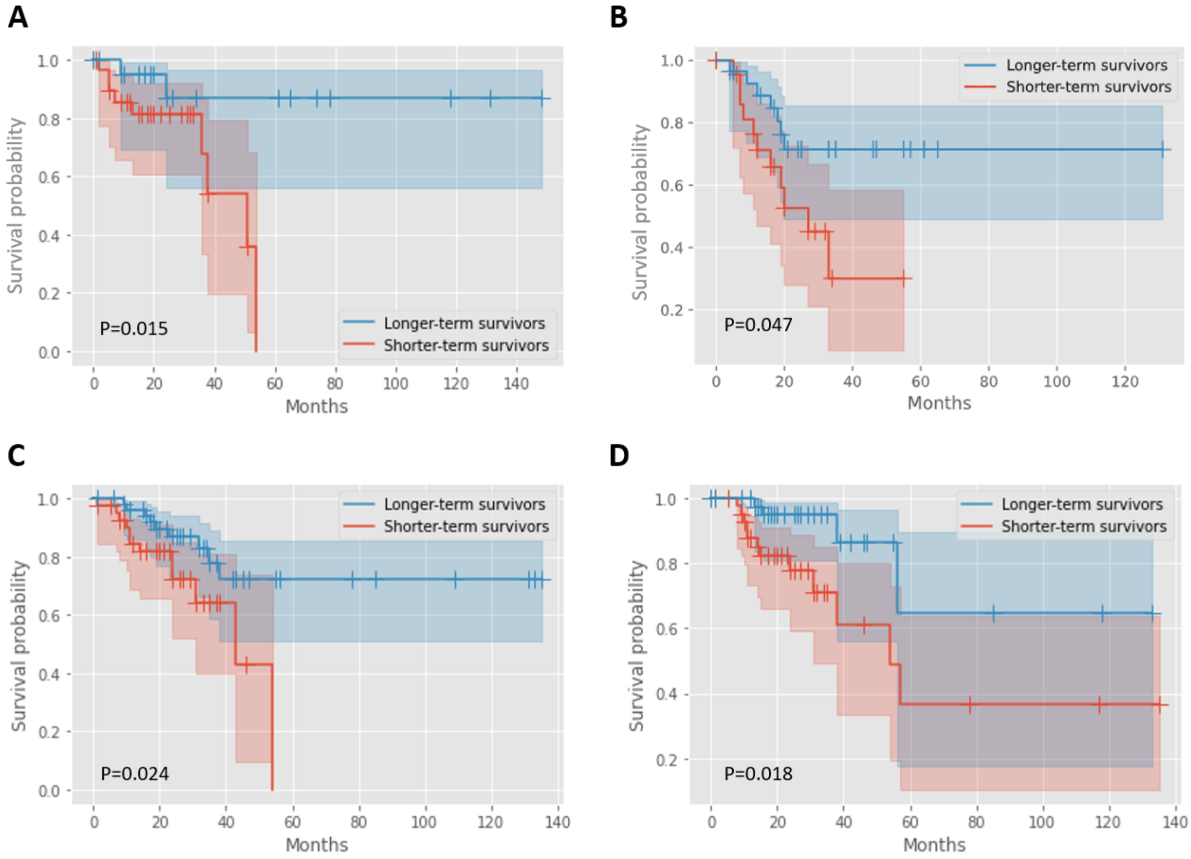
A
Survival probability
Months
P=0.015
Longer-term survivors
Shorter-term survivors
B
Survival probability
Months
P=0.047
Longer-term survivors
Shorter-term survivors
C
Survival probability
Months
P=0.024
Longer-term survivors
Shorter-term survivors
D
Survival probability
Months
P=0.018
Longer-term survivors
Shorter-term survivors

Supplementary Figure 2. MOMA characterized pathology imaging signals correlated with the expression level of the *BECN1* gene. (A) MOMA identified a moderate association between histopathology imaging and the expression level of *BECN1*. Performance in the TCGA held-out test set was shown. (B) We successfully validated the MOMA-based prediction model in the Nurses' Health Study and Health Professionals Follow-Up Study cohorts, demonstrating the validity of the identified associations. (C) Attention visualization of *BECN1*-low histopathology images. (D) Attention visualization of *BECN1*-high histopathology images. Regions of cancer cells received a high attention level from the *BECN1* prediction model. Both mucosa and tumor receive high attention weights in BECN1 prediction. In *BECN1*-low, the model pays more attention to the cancer-associated stroma, but in *BECN1*-high, the model focuses more on lymphocytes. MUC: mucus; TUM: colorectal adenocarcinoma epithelium; STR: cancer-associated stroma; LYM: lymphocytes.

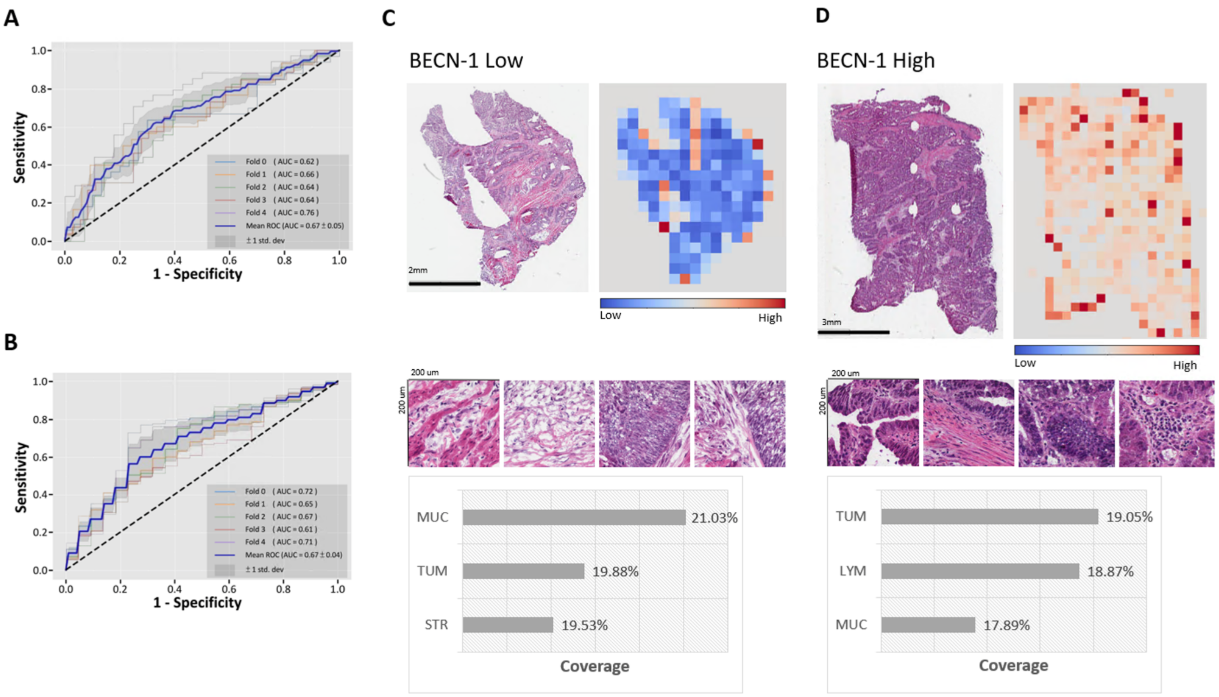
A
Sensitivity
1 - Specificity
Fold 0 (AUC = 0.62)
Fold 1 (AUC = 0.66)
Fold 2 (AUC = 0.64)
Fold 3 (AUC = 0.64)
Fold 4 (AUC = 0.76)
Mean ROC (AUC = 0.67 ± 0.05)
± 1 std. dev
B
Sensitivity
1 - Specificity
Fold 0 (AUC = 0.72)
Fold 1 (AUC = 0.65)
Fold 2 (AUC = 0.67)
Fold 3 (AUC = 0.61)
Fold 4 (AUC = 0.71)
Mean ROC (AUC = 0.67 ± 0.04)
± 1 std. dev
C
BECN-1 Low
2mm
Low
High
200 um
200 um
MUC 21.03%
TUM 19.88%
STR 19.53%
Coverage
D
BECN-1 High
3mm
Low
High
200 um
200 um
TUM 19.05%
LYM 18.87%
MUC 17.89%
Coverage

Supplementary Figure 3. MOMA identified the association between *BRAF* c.1799T>A (p.V600E) mutation and histopathology image patterns. (A) MOMA characterized a moderate correlation between *BRAF* c.1799T>A (p.V600E) mutation and histopathology image features. Results from the TCGA held-out test set were shown. (B) Attention visualization of a histopathology image from a *BRAF* wild-type patient. (C) Attention visualization of a histopathology image from a *BRAF* c.1799T>A (p.V600E) mutation patient. Regions of muscle, stroma, cancers, and mucus received high attention in this molecular classification task. TUM: colorectal adenocarcinoma epithelium; STR: cancer-associated stroma; MUC: mucus; MUS: smooth muscle.

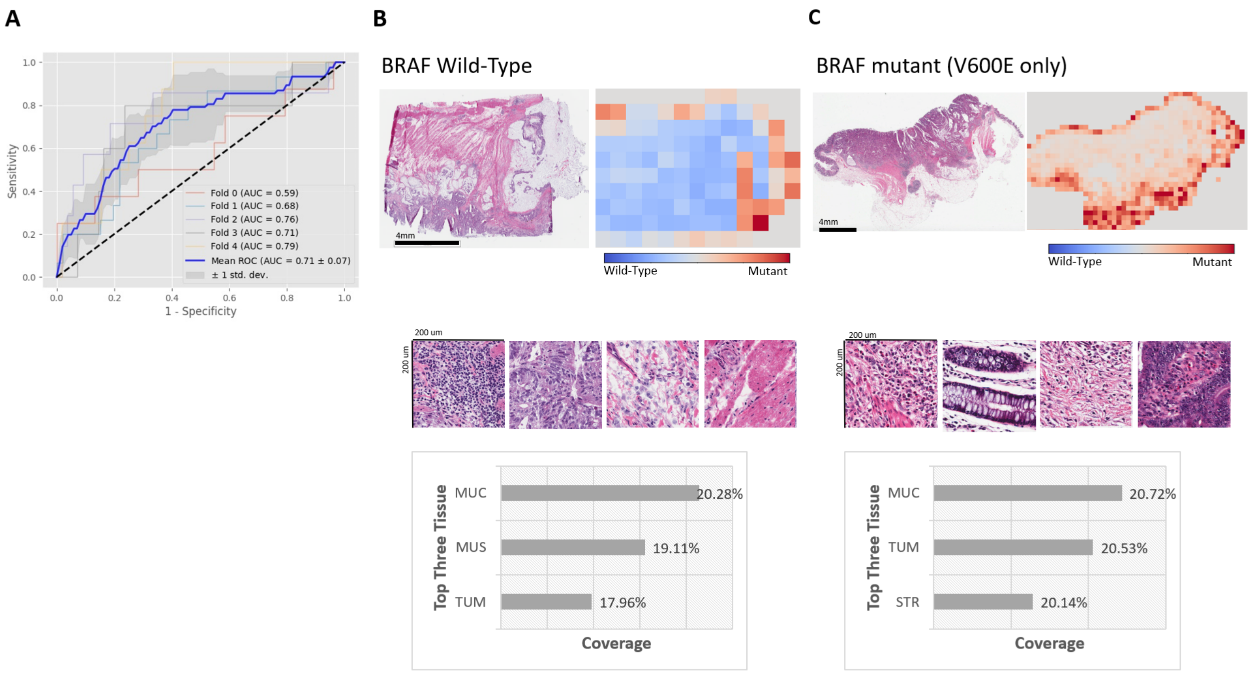

Supplementary Figure 4. MOMA identified the association between *BRAF* mutations at any loci and histopathology image patterns. (A) MOMA characterized a moderate correlation between *BRAF* mutation and histopathology image features. Results from the TCGA held-out test set were shown. (B) The same model generated by MOMA was validated in the Nurses' Health Study and Health Professionals Follow-Up Study cohorts. (C) Attention visualization of a histopathology image from a BRAF wild-type patient. (D) Attention visualization of a histopathology image from a BRAF-mutated patient. Regions of stroma, cancers, and mucus received high attention in this molecular classification task. STR: cancer-associated stroma; MUC: mucus; TUM: colorectal adenocarcinoma epithelium.

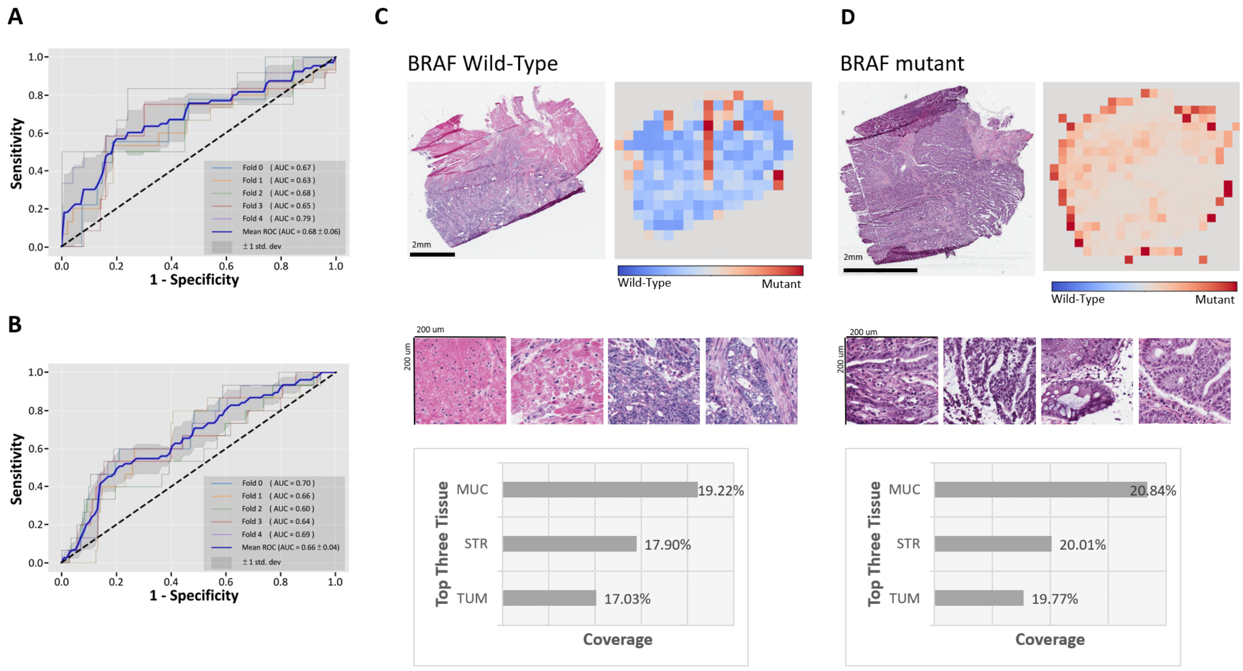

Supplementary Figure 5. MOMA weakly predicts overexpression of the *HIF1A* gene using histopathology images. (A) Performance in the TCGA held-out test set was shown. (B) The results are validated in the Nurses' Health Study and Health Professionals Follow-up Study cohorts.

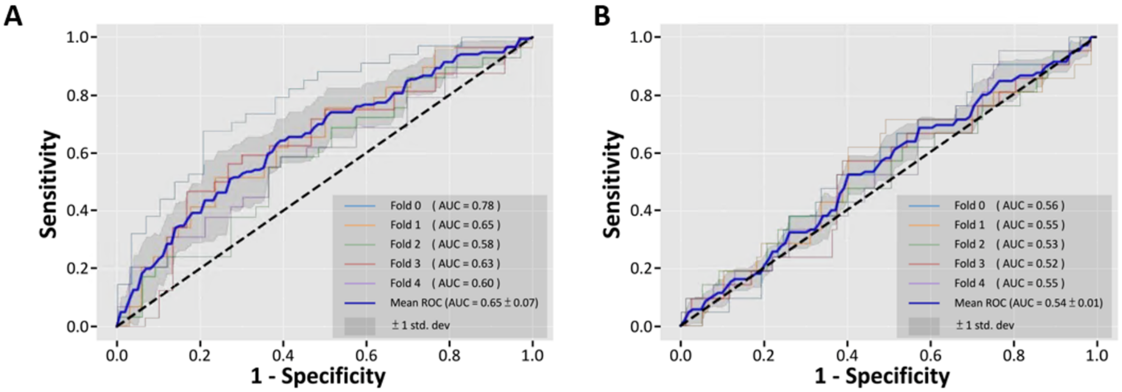

Supplementary Figure 6. MOMA identified a moderate association between histopathology images and *PIK3CA* mutation status. (A) Performance in the TCGA held-out test set was shown. (B) The results are validated in the Nurses' Health Study and Health Professionals Follow-up Study cohorts.

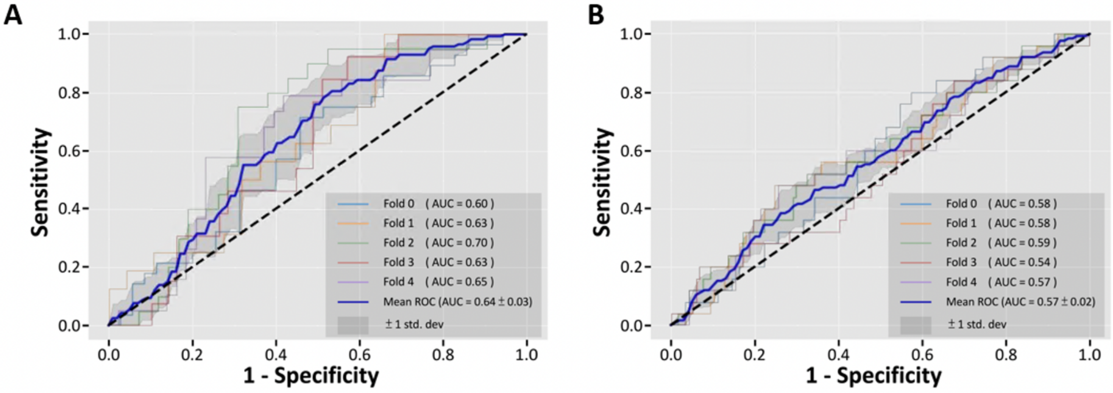

Supplementary Figure 7. MOMA associates histopathology image patterns with the CpG Island Methylator Phenotype (CIMP). (A) CIMP prediction performance in the TCGA held-out test set. (B) Our CIMP prediction model was validated in the Nurses' Health Study and Health Professionals Follow-up Study cohorts. (C) Attention visualization of non-CIMP-high histopathology images. (D) Attention visualization of CIMP-high histopathology images. TUM: colorectal adenocarcinoma epithelium; STR: cancer-associated stroma; MUC: mucus; LYM: lymphocytes.

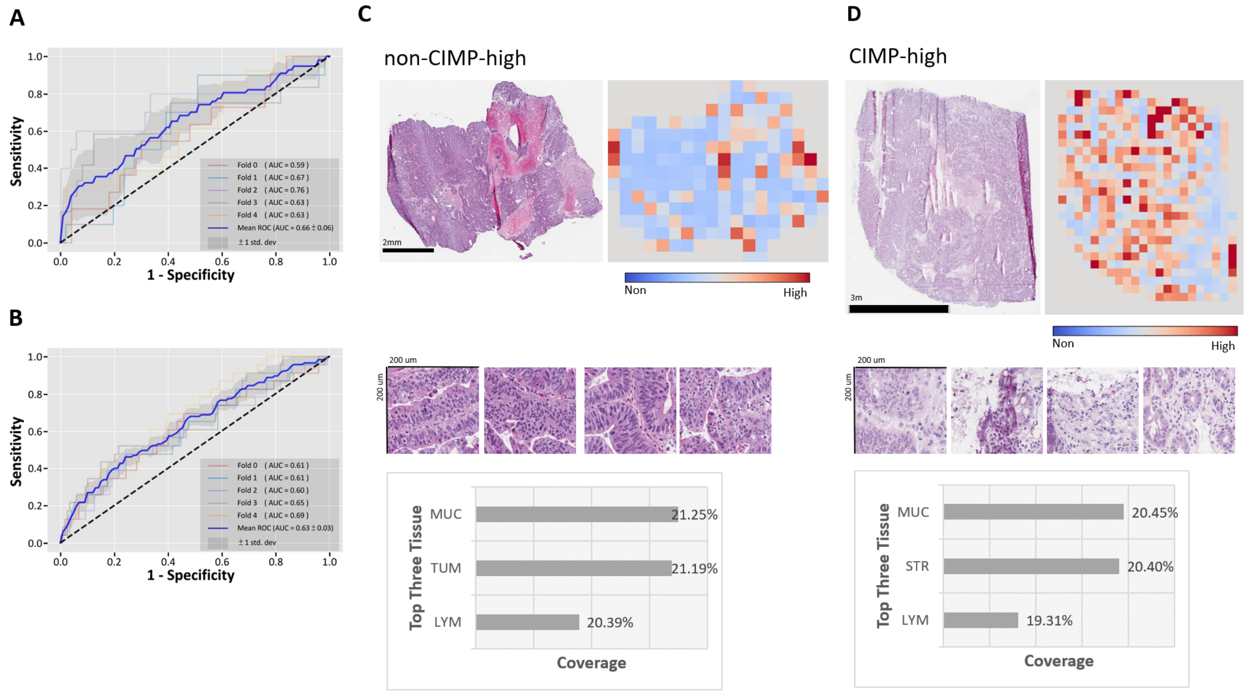

Supplementary Figure 8. MOMA identified the association between CMS and histopathology image patterns. (A) MOMA characterized a moderate correlation between CMS2 and CMS4 in histopathology image features. Results from the TCGA held-out test set were shown. (B) Attention visualization of a histopathology image from a CMS2 patient. (C) Attention visualization of a histopathology image from a CMS4 patient. Regions of stroma, cancers, lymphocytes, and mucus received high attention in this molecular classification task. MUC: mucus; TUM: colorectal adenocarcinoma epithelium; STR: cancer-associated stroma; LYM: lymphocytes.

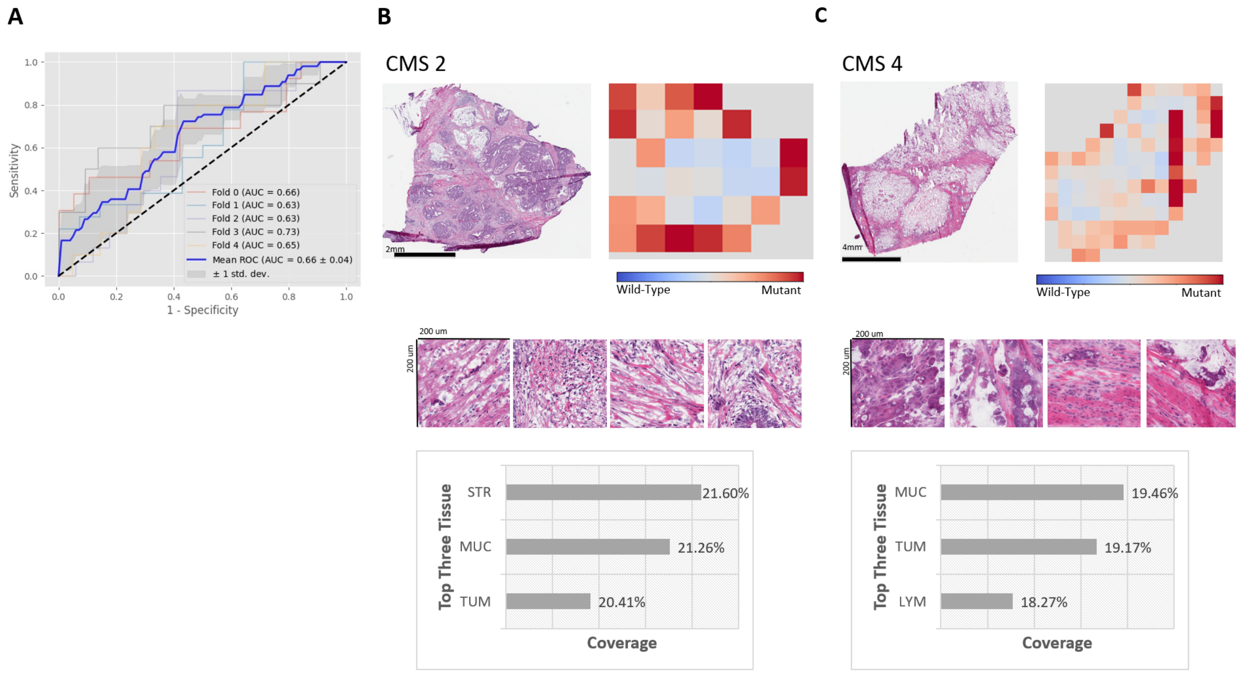

Supplementary Table 1. Additional model performance metrics for multi-omics characterization via histopathology image analyses.

	Dataset	Accuracy	Precision	Sensitivity (i.e., Recall)	Specificity	AUROC
Microsatellite Instability	TCGA	0.80	0.75	0.89	0.75	0.88
	NHS-HPFS	0.76	0.67	0.86	0.57	0.76
BRAF Mutation c.1799T>A (p.V600E)	TCGA	0.67	0.63	0.78	0.61	0.71
	NHS-HPFS	Mutation Loci Not Available				
BECN1 Overexpression	TCGA	0.60	0.58	0.73	0.61	0.67
	NHS-HPFS	0.85	0.83	0.67	0.64	0.67
CpG Island Methylator Phenotype	TCGA	0.77	0.65	0.68	0.55	0.66
	NHS-HPFS	0.68	0.63	0.67	0.53	0.63
Consensus Molecular Subtypes	TCGA	0.69	0.86	0.73	0.57	0.66
	NHS-HPFS	Transcriptomic Data Not Available				

* Gene names are italicized

Supplementary Table 2. Performance comparison between MOMA, a patch-based standard convolutional neural network, and a previously published method (Kather et al.) in MSI prediction (two-sided Wilcoxon signed-rank test).

	MOMA	Patch-based	Kather et al.
Fold 1	0.92	0.85	-
Fold 2	0.92	0.87	-
Fold 3	0.79	0.78	-
Fold 4	0.94	0.94	-
Fold 5	0.89	0.85	-
Mean AUROC	0.88	0.85	0.84
Two-sided Wilcoxon Signed-Rank Test P-Value	-	Not significant	

Supplementary Table 3. Performance comparison between MOMA and PC-CHiP in copy number variation prediction.

	Gene	P-value
Deletion in Colon Cancer	FAT1	Not significant
	PPP2R2A	3.08E-07
	FHIT	7.21E-62
	PTEN	Not significant
	LINC00290	1.80E-136
	MACROD2	Not significant
	CSMD1	Not significant
Amplification in Colon Cancer	BCL2L1	2.71E-168
	ZNF217	Not significant
Deletion in Rectal Cancer	PPP2R2A	2.34E-87
	MACROD2	2.06E-28
	CSMD1	6.15E-54

Supplementary Table 4. Performance of whole-genome doubling prediction of MOMA compared with that of PC-CHiP (two-sided Wilcoxon signed-rank test).

	Colon Cancer		Rectal Cancer	
	MOMA	PC-CHiP	MOMA	PC-CHiP
Area Under the Receiver Operating Characteristic Curve	0.72	0.65	0.63	0.51
Two-sided Wilcoxon Signed Rank Test P-Value	Not significant		5.12E-19	
