# Supplementary material for: Histopathology images predict multi-omics aberrations and prognoses in colorectal cancer patients
Source: Nat Commun. 2023 Apr 13;14:2102. doi: 10.1038/s41467-023-37179-4 (PMC10102208; doi:10.1038/s41467-023-37179-4)
Supplement: Supplementary file 4 — Description of Additional Supplementary Files [file 41467_2023_37179_MOESM4_ESM.docx]

**Description of Additional Supplementary Files**

Supplementary Data 1

Description: Performance metrics of histopathology-based multi-omics characterization and survival prediction stratified by colon and rectal cancers.
